# Phage vs. Phage: Direct Selections of Sandwich Binding Pairs

**DOI:** 10.3390/v15030807

**Published:** 2023-03-22

**Authors:** Emily C. Sanders, Alicia M. Santos, Eugene K. Nguyen, Aidan A. Gelston, Sudipta Majumdar, Gregory A. Weiss

**Affiliations:** 1 Departments of Chemistry, University of California, Irvine, CA 92697, USA; 2 Departments of Molecular Biology and Biochemistry, University of California, Irvine, CA 92697, USA; 3 Departments of Pharmaceutical Sciences, University of California, Irvine, CA 92697, USA

**Keywords:** phage display, sandwich binding, biomarker diagnostics, enzyme-linked immunosorbent assay (ELISA)

## Abstract

The sandwich format immunoassay is generally more sensitive and specific than more common assay formats, including direct, indirect, or competitive. A sandwich assay, however, requires two receptors to bind non-competitively to the target analyte. Typically, pairs of antibodies (Abs) or antibody fragments (Fabs) that are capable of forming a sandwiching with the target are identified through a slow, guess-and-check method with panels of candidate binding partners. Additionally, sandwich assays that are reliant on commercial antibodies can suffer from changes to reagent quality outside the researchers’ control. This report presents a reimagined and simplified phage display selection protocol that directly identifies sandwich binding peptides and Fabs. The approach yielded two sandwich pairs, one peptide–peptide and one Fab–peptide sandwich for the cancer and Parkinson’s disease biomarker DJ-1. Requiring just a few weeks to identify, the sandwich pairs delivered apparent affinity that is comparable to other commercial peptide and antibody sandwiches. The results reported here could expand the availability of sandwich binding partners for a wide range of clinical biomarker assays.

## 1. Introduction

Precision medicine promises a future of individually tailored diagnostics and treatments. Achieving this degree of medical personalization will likely require a large number of sensitive, accurate, biomarker-based diagnostics to detect nascent disease and to monitor responses to treatments [1,2]. As this approach requires frequent testing, samples ideally would be collected noninvasively or in minimal quantities through a “liquid biopsy” (e.g., by measuring biomarkers in blood, urine, or saliva) [3,4,5,6]. Challenges to the approach include the variance in the composition of some physiological fluids and also the requirement to detect biomarkers in low concentrations [7,8,9,10,11]. Thus, precision medicine necessitates the development of platforms for sensitive and selective quantitation of biomarkers within complex physiological fluids. For example, the experiments reported here focus on the detection of protein deglycase 1 (DJ-1), a multi-functional biomarker for several cancers and also for Parkinson’s disease [12,13,14,15,16,17,18].

By providing two receptors for the target antigen, the sandwich-format assay can address the dual challenges of sensitive and selective binding [19,20,21,22,23,24]. However, this assay format requires two compatible receptors to noncompetitively and simultaneously bind to the same antigen. The most widely deployed example of a sandwich-format assay is the at-home pregnancy test, which detects the early pregnancy hormone human chorionic gonadotropin [25]. The sandwich-format assay has also been applied to many other diagnostics, including for bladder cancer [26], severe skeletal injury [27], and heart failure [28]. The requirement for the target to bridge both receptors addresses the specificity challenge inherent in biological assays. Additionally, an improvement in sensitivity is often observed through conversion of assays to the sandwich format.

Unfortunately, identification of antibody pairs for sandwich format assays is often cumbersome, slow, and costly. After acquisition of the available commercial antibodies, each pair of antibodies must be screened for noncompetitive binding interactions with the target. As the number of testable antibodies increases, the number of required assays increases exponentially. Furthermore, an effective assay with a commercial sandwich pair can be lost through product discontinuation or drift in either quality control or the genetics of the hybridoma cell line [29,30].

To address these issues, several reports describe methods for improved sandwich pair discovery. For example, Ki et al. immobilized cetuximab, a commercially available antibody, to a microtiter plate and performed antibody phage display selections [31]. While this technique successfully identified a sandwich pair, the method still requires a commercial antibody bound to solid support. Gorman et al. pioneered a powerful phage-display technique for the direct selection of monobody sandwich pairs, Megaprimer Shuffling for Tandem Affinity Reagents (MegaSTAR). With MegaSTAR, the researchers identified high affinity pairs of sandwiched nanobodies [32]. However, the approach requires lengthy and potentially challenging subcloning steps to generate a tandem phage library for each target. Therefore streamlined and easy approaches to discovering sandwich binding pairs remains an unsolved, important challenge.

**Figure 1:**
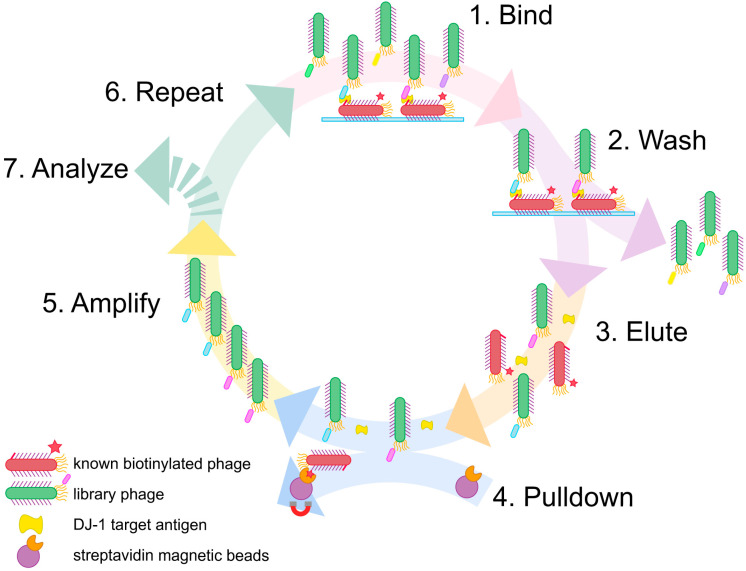
Phage vs. phage selection strategy. (1) Biotinylated phage displaying the known binding partner are adsorbed to a microtiter plate and exposed to the target. Next, the phage library is added and allowed to bind. (2) Nonbinding phages are removed by washing. (3) Sandwich assemblies are eluted from the microtiter plate by treatment with low pH solution and sonication. (4) In the eluate, biotinylated phage display with the known binding partner can removed from the solution through binding to magnetic streptavidin beads. (5) The sandwich forming pair of phages are used to infect *E. coli* and amplified. (6) The process is repeated with the amplified selectants as the phage library for the next round of selection. (7) After three to four rounds of selection, the selectants are identified by DNA sequencing.

Here we address this challenge with a simple, expedient, and generalizable selection method, termed phage vs. phage (PvP) (Figure 1). Using PvP, non-overlapping phage-displayed binders specific for DJ-1 were identified from both peptide and antibody fragment (Fab) libraries. Enzyme-linked immunosorbent assays (ELISAs) demonstrate the specific and sensitive sandwich binding pairs that are generated by the method.

## 2. Results and Discussion

Sandwich phage selections. The method reported here builds on conventional phage display. First, conventional selections identify an effective binder for one half of the sandwich assembly, which becomes the “known binding partner” for subsequent steps. Next, the phages displaying the known binding partner are biotinylated and adsorbed to a microtiter plate; the usual blocking step then prevents nonspecific interactions. The target antigen is added and allowed to bind to the surface-adsorbed, phage-displayed known binder. The phage-displayed protein or peptide library is added next. If the target antigen is present in excess quantities, only noncompetitive binding partners that are capable of forming a sandwich interaction with the known binding partner will be selected.

This approach has a major problem, however. Phages displaying the known binding partner will appear in overwhelming abundance compared to any selectant from the library. Such phages must be exhaustively culled from the eluted phage following each round to avoid their amplification; without this step, selectants from each round become suppressed amidst excess phages displaying the known binding partner to the target antigen. We demonstrate in this report that these undesirable phages can be biotinylated before selection and then removed using streptavidin magnetic beads before the phage amplification step. This strategy was inspired by the streptavidin-biotin immunoprecipitation assay and is made possible by the strong, functionally irreversible interaction between biotin and streptavidin [33,34].

Here, the PvP approach was used to discover a sandwich binding pair to the target DJ-1. A dimeric, globular protein such as DJ-1 is an ideal target for this approach. The surfaces of such proteins are generally comprised of multiple “pockets” where ligands can bind via weaker, noncovalent interactions [35]. A previously reported phage-displayed peptide, termed DPep1Φ (where the symbol Φ denotes phage), was chosen as the known binding partner for PvP selections with DJ-1 [36].

**Figure 2:**
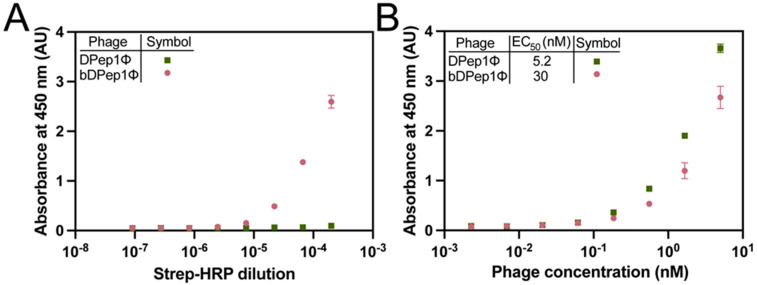
Efficient phage biotinylation and its effects on target binding. (**A**) Biotinylation of phages (bDPep1Φ) was confirmed via direct detection ELISA with peroxidase-conjugated streptavidin (Strep-HRP) assay. (**B**) An indirect phage ELISA with DJ-1 verified that both biotinylated and non-biotinylated page (DPep1Φ) could bind the target antigen despite biotinylation. ELISA data were fit to a four-parameter logistic curve. Error bars indicate standard deviation (*n* = 3); each data point includes error bars, although most are quite small. HRP = horseradish peroxidase.

First, DPep1Φ were subjected to biotinylation through treatment with N-hydroxysuccinimide biotin to yield biotinylated DPep1Φ, abbreviated here as bDPep1Φ. A direct ELISA, in which an enzyme-conjugated receptor (horse radish peroxidase conjugated to streptavidin, termed Strep-HRP) binds to a plate-bound antigen (bDPep1Φ), assessed the success of the biotinylation reaction (Figure 2A). The bDPep1Φ dose-dependent response with Strep-HRP, as compared to a non-biotinylated phage control, demonstrated successful biotinylation. Importantly, an indirect phage ELISA (defined here as an assay in which microtiter plate-coated antigens are bound by antigen-specific phage and detected with an anti-phage antibody) demonstrated that bDPep1Φ retains effective binding to plate-bound DJ-1 (Figure 2B). Each phage particle (MW ≈ 16.5 MDa) has thousands of capsid proteins each with N-terminal and Lys amines available for NHS ester modification; as shown in Figure 2B, capsid protein-free amines must outnumber potential modification sites within the displayed peptide, as the interaction between bDPep1Φ and DJ-1 remains strong, despite biotinylation.

The PvP strategy requires essentially complete removal of biotinylated DPep1Φ from the eluted solution. A pull-down titration assay with bDPep1Φ and streptavidin-coated magnetic beads determined the bead concentration (0.389 mg/mL) required to remove 17 nM biotinylated phage from solution (UV absorbance assay shown in Figure S1). To account for the relatively high detection limits of the UV-Vis absorbance assay, the streptavidin-coated magnetic beads were used at a higher concentration (2.00 mg/mL) during selections to ensure complete elimination of bDPep1Φ after each round of selection.

**Figure 3:**
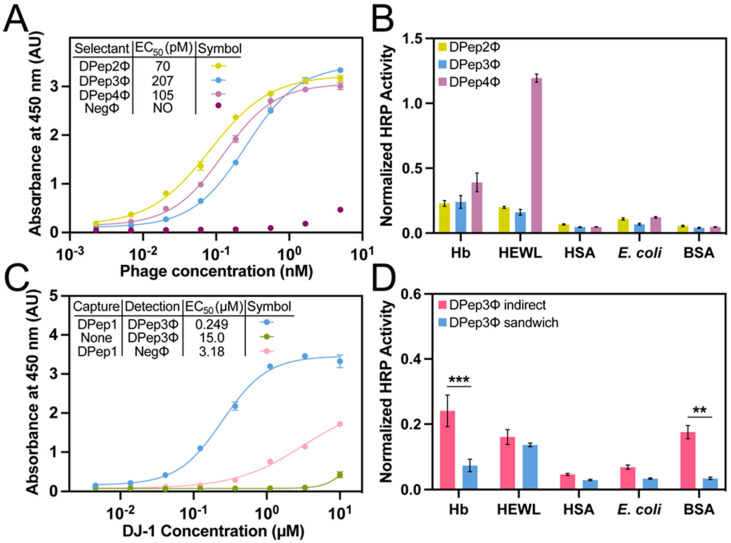
Sensitivity and specificity determination of sandwich peptide selectants. (**A**) A dose-dependent direct phage assay with sandwich peptide selectants binding to DJ-1. Sub-micromolar phage EC_50_ values were observed. NO = not observed. (**B**) An indirect phage assay with Hb, HEWL, HSA, *E. coli* lysate, and BSA determined the specificity of the sandwich selectants. Data are normalized to the DJ-1 signal. (**C**) Streptavidin-bound DPep1 peptide assay for sandwiching the target DJ-1 with DPep3Φ. The resultant dose-dependent response yielded an EC_50_ for binding to DJ-1 of 249 pM. (**D**) The DPep1-DPep3Φ sandwich interaction also demonstrated the interaction’s improved selectivity for DJ-1. ANOVA with Sidak’s multiple comparisons yielded *p*-values of <0.01 (**) and <0.001 (***). ELISA data were fit to a four-parameter logistic curve. Error bars, included for every data point, depict standard error of the mean (SEM) (*n* = 3).

Characterization of sandwich peptide selectants. The PvP selection strategy was first performed with a phage P8-displayed, mega-random peptide library [37] (Table S1). The method yielded eight unique phage-displayed peptides, three of which could be produced in sufficient quantities for further characterization (Table 1). The phage indirect ELISA format performed with DJ-1 and the selectants (DPep2Φ, DPep3Φ, and DPep4Φ) yielded sigmoidal curves that are characteristic of dose-dependent binding (Figure 3A). The selectants demonstrated sub-micromolar phage EC_50_ values (0.70–2.07 nM), which are similar to the EC_50_ for DPep1Φ (5.2 nM). Despite comparable apparent binding affinities, the PvP selectants are not homologous to DPep1 in either amino acid sequence or theoretical isoelectric point (pI) (Table 1). These sequence data suggest the peptides that were identified by PvP are distinctly different from DPep1 and interact with DJ-1 noncompetitively. In addition to the indirect ELISA described here, the experiments below demonstrated formation of a sandwich binding interaction for binding to DJ-1.

The relative specificity of each PvP selectant was determined by indirect phage ELISA with a profile of competing antigens (Table S2, Figure 3B). Hemoglobin (Hb), human serum albumin (HSA), and F^-^ *E. coli* lysate are potential interfering substances in biofluids (e.g., in urine). The high-pI hen egg white lysozyme (HEWL) was chosen to assess non-specificity to the anionic phage coat proteins. Due to its ubiquity as a blocking agent in assays, bovine serum albumin (BSA) was also included. Compared to the other PvP selectants, DPep4Φ had less specificity for DJ-1 compared to HEWL. This assay can also test for improvement in both sensitivity and specificity from the sandwich format assay, as described in the next section.

Sandwich ELISAs with peptide PvP selectants. Next, we evaluated sandwich binding between DPep1 and the PvP selectants. For this experiment, non-phage-displayed, biotinylated DPep1 was chemically synthesized. This peptide was then immobilized on a streptavidin-coated microtiter plate. This layer was used to capture DJ-1, which was added in serial dilutions during a 10-min incubation. The addition of phage peptide PvP selectants completed the sandwich assembly and, lastly, a peroxidase-conjugated anti-phage antibody quantified the binding interaction (Figure 3C). In this format, the DPep2Φ and DPep4Φ sandwich ELISAs failed to produce a significant signal over the background; these two peptides likely were incapable of forming a sandwich interaction. However, the DPep3Φ sandwich ELISA returned a sigmoidal binding curve and an EC_50_ that is comparable to a previously reported sandwich phage/antibody ELISA with a commercial anti-DJ-1 antibody [36]. At DJ-1 concentrations above 1 µM, some non-specificity with negative control phage (NegΦ) is observed; however, these high concentrations are unlikely to occur in biofluids.

The sandwich strep-DPep1/phage ELISA with the panel of interfering antigens—hemoglobin (Hb, HEWL), HSA, *E. coli* lysate, and BSA—evaluated the specificity improvements with the sandwich assembly (Figure 3D). Since the assay formats (indirect vs. sandwich) are not analogous, the data were normalized to the DJ-1 signal for each assay format. Statistical analysis of these normalized data revealed a substantial and statistically significant enhancement of specificity; the nonspecific Hb and BSA signal dropped significantly in the sandwich format (*p* < 0.001 and <0.01, respectively). As expected, background binding to HEWL, which has a high pI causing it to bind non-specifically to the phage surface [38], was unaffected by the sandwich format. These data indicate that the PvP selection strategy can yield sandwich binding peptides with improved selectivity compared to the equivalent single phage binding partners.

**Figure 4:**
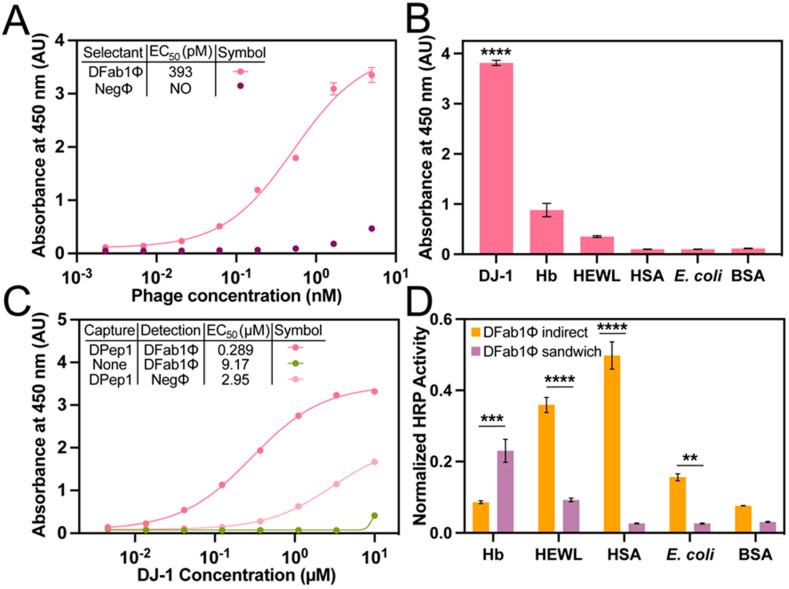
Sensitivity and specificity determination of DFab1. (**A**) A dose-dependent direct phage assay with DFab1Φ and NegΦ. Data were fit to a four-parameter logistic curve and yielded picomolar EC_50_ values for the DFab1Φ interaction with DJ-1. Error bars represent SEM (*n* = 3). NO = not observed. (**B**) An indirect phage assay with DJ-1, Hb, HEWL, HSA, *E. coli* lysate, and BSA determined the specificity of the sandwich selectants. ANOVA with Tukey’s multiple comparisons yielded *p*-values of <0.0001 (****). (**C**) A similar dose-dependent response with an EC_50_ of 1.53 μM was observed in an identical sandwich ELISA with DFab1Φ. (**D**) The DPep1-DFab1Φ also demonstrated strong selectivity for DJ-1. ELISA data were fit to a four-parameter logistic curve. Data are normalized to DFab1Φ binding to DJ-1. Error is represented as SEM (*n* = 3). NO = not observed. ANOVA with Tukey’s multiple comparisons yielded *p*-values of <0.01 (**), <0.001 (***), <0.0001 (****).

PvP selections with a Fab phage-displayed library. Selections with a Fab phage library demonstrated the generalizability of the PvP method. The procedure was performed exactly as described above using bDPep1Φ, but with a P3 Fab phage-displayed library in place of the peptide library (Table S3) [39]. Three rounds of PvP selections identified a DJ-1 binding, phage-displayed Fab (DFab1Φ) (Table 2). An indirect phage ELISA with immobilized DJ-1 revealed a DFab1Φ EC_50_ of 393 pM, a value of similar magnitude to the peptides that were isolated from PvP selectants (Figure 4A). Similarly, DFab1Φ demonstrated high selectivity for DJ-1 versus the panel of interfering antigens (Figure 4B). Thus, the PvP selection strategy is generalizable and can be performed with a variety of phage libraries.

The previously described sandwich strep-DPep1/phage ELISA had an EC_50_ for binding to DJ-1 of 289 nM (Figure 4C). The DPep1/DFab1Φ sandwich assembly demonstrated improved specificity for DJ-1 compared to the indirect phage ELISA for HEWL, HSA, and *E. coli*. Interestingly, a slight decrease in specificity over DJ-1 for Hb was also observed (Figure 4D), but homology searches did not reveal any significant similarities between these proteins that could explain the origin of this non-specificity. These data, when considered with the dose-dependent sandwich ELISA data, demonstrate that the PvP selection strategy can yield sandwich binding peptides that can rival a commercial antibody sandwich in sensitivity and improve selectivity compared to the individual phage binding partners.

## 3. Conclusions

The sandwich ELISA format is widely deployed in healthcare due to its high sensitivity and specificity, but identification of “matched” or noncompetitive binding sandwich pairs beyond a “guess-and-check” method remains elusive. Furthermore, even when a sandwich assay is established with a commercial antibody, the assay’s efficacy can be compromised by circumstances beyond the researchers’ control. This report presents a modified phage display protocol that directly selects for peptides and Fabs that bind to DJ-1 in concert with the well-established DPep1. Primary selectants, not subject to further affinity maturation, demonstrated binding capabilities that are comparable to a similar sandwich assay with a commercial antibody and improved specificity over the corresponding indirect phage ELISA. Additionally, PvP selections are comparatively quite simple to execute and can be implemented in any laboratory currently performing traditional phage display selections. Thus, the PvP selection method could provide the basis for expanding applications of sandwich assays in clinical settings.

## 4. Materials and Methods

Materials. Unless otherwise specified, reagents were sourced from Sigma Aldrich. Antibody-fragment phage libraries were generously gifted from the laboratory of Prof. Sachdev Sidhu. DJ-1 protein was expressed and purified as previously described [36].

Phage propagation and purification. The phagemid DNA was transformed into SS320 competent *E. coli*, and cells were plated on an LB agar plate supplemented with 50 μg/mL carbenicillin before incubation overnight at 37 °C. A single colony was selected to inoculate 25 mL of 2YT (autoclaved solution of 16 g tryptone, 5 g NaCl, 10 g yeast extract in 1 L final volume water) supplemented with 50 μg/mL carbenicillin and 2.5 μg/mL tetracycline. The culture was shaken at 37 °C until its OD_600_ reached 0.5; then, 30 μM IPTG and sufficient M13KO7 to achieve a multiplicity of infection of 4.6 was added. After an additional 45 min incubation, 8 mL of the culture was used to inoculate 150 mL of 2YT supplemented with carbenicillin (50 μg/mL), kanamycin (20 μg/mL), and IPTG (30 μM). This culture was incubated at 30 °C with shaking at 225 rpm for 18 h. 

The cultures were centrifuged at 10 krpm (15,300× *g*) for 10 min. The supernatant was transferred to a centrifuge tube containing 1/5 of the supernatant’s volume of a mixture of PEG8000 (20%, *w*/*v*) and NaCl (2.5 M). The tube was inverted five times to mix and incubated on ice for 30 min. The solution was centrifuged at 10 krpm (15,300× *g*) for 15 min. The supernatant was decanted, and the tubes were centrifuged for an additional 4 min at 4 krpm (2429× *g*). The pellets were resuspended in PBS and the precipitation steps were repeated. Phage concentrations were quantified by measuring absorbance at 268 nm. Finally, the phages were diluted to 60 nM, flash frozen with glycerol (10%, *v*/*v*), and stored at −80 °C.

NHS-biotin modification of phage. M13 bacteriophages (DPep1Φ) were diluted to 1 mg/mL. The diluted phage (1 mL) and EZ-Link NHS-biotin (ThermoFisher, Waltham, MA, USA, 33 mM in DMSO) were combined and incubated overnight at room temperature. After incubation, the solution was added to a 3000 MWCO membrane and dialyzed with stirring in 1 L of PBS. After 4 h, the PBS was replaced with fresh solution and the dialysis was continued overnight.

Direct strep-HRP ELISA. Unless otherwise specified, all incubation steps were performed at 150 rpm and room temperature. A 96-well Maxisorp plate was coated with 100 μL/well of phage (1 nM in 50 mM Na_2_CO_3_) and incubated overnight at 4 °C. The coating solution was discarded, and the wells were blocked with 400 μL of blocking buffer (0.2% BSA in PBS) for 30 min. The blocking solution was removed, and the wells were washed three times with PBS-T (0.05% Tween-20 in PBS) followed by incubation for 1 h with serially diluted HRP-conjugated streptavidin (ThermoFisher, Waltham, MA, USA) in PBT (0.2% BSA, 0.05% Tween-20 in PBS). The solution was again discarded, and the wells were washed five additional times with PBS-T and one final time with PBS. 1-Step Ultra TMB-ELISA Substrate Solution (ThermoFisher, Waltham, MA, USA, 100 μL per well) was added to each well, followed by 2 M H_2_SO_4_ (100 μL) after sufficient signal had developed. The absorbance at 450 nm was measured with an Epoch Microplate Spectrophotometer (BioTek, Charlotte, NC, USA) and the resulting data were analyzed and fit using GraphPad Prism 9. 

Dynabeads titration. Dynabeads M-270 Streptavidin (Invitrogen, Waltham, MA, USA) were serially diluted in PBS from 4.44 to 0.389 mg/mL. Solutions of phage were added to the Dynabead dilutions such that the final phage concentration was 17 nM. The solutions were incubated with shaking at 150 rpm for 10 min. A magnet was used to isolate the Dynabeads and the supernatant was pipetted into a new container. Negative control solutions with no phage were also performed with identical conditions. The final phage concentration was determined by absorbance at 268 nm.

Indirect phage affinity ELISA. A 96-well Nunc Maxisorp plate was coated with 10 μg/mL DJ-1 in coating buffer (100 μL/well, 50 mM Na_2_CO_3_) and incubated overnight at 4 °C. The coating solution was discarded, and the plate was blocked and washed as previously described. The plate was incubated with serially diluted phage in PBT for 1 h. The solution was removed, and 100 μL of 1:5000 Anti-M13 Monoclonal Antibody conjugated to HRP (Creative Diagnostics, New York City, NY, USA) diluted in PBT was added to each well. The plate was incubated for another 30 min, the antibody solution was discarded, and the wells were washed five times with PBS-T and once with PBS. Then, the plate was treated as described above.

Phage vs. Phage Selections. All incubation steps were performed on an orbital shaker at 150 rpm and room temperature. Biotinylated DPep1Φ (bDPep1Φ) was diluted to 1 nM in coating buffer and incubated on a Nunc MaxiSorp plate overnight (100 μL/well). The solution was discarded, and the wells were blocked for 30 min with 400 μL of either casein, BSA, HSA (0.2%), or Pierce Protein-Free Blocking Buffer. Next, the wells were washed with 100 μL PBS-T three times and incubated with 100 μL DJ-1 (3 μM in PBS with 0.05% Tween-20 and 0.2% corresponding blocking agent, or in Piece Protein-Free Blocking Buffer) for 1 h. The wells were again washed three times and incubated with the prepared phage library (60 nM in blocking buffer, 100 μL per well) for 90 min. The wells were washed again three times to remove nonspecifically binding, phage-displayed ligands.

The sandwich assemblies were eluted from the plate with HCl (0.1 M, 100 μL per well) and sonication in a water bath for 10 min. The eluted solutions were combined and neutralized with 1/3 the volume of Tris-HCl (1 M, pH 8.0). Dynabeads M-270 Streptavidin were added (2 mg/mL) and the solution was incubated at 4 °C with shaking at 150 rpm for 30 min. The magnetic beads were isolated with a magnet and the supernatant was collected. The eluted phage solution was used to infect log phase *E. coli* XL-1 Blue cells (20 mL, supplemented with 5 μg/mL tetracycline). The culture was incubated with shaking at 225 rpm at 37 °C for 1 h. Next, M13KO7 helper phage (NEB) was added to reach a multiplicity of infection of 4.6 and the culture was incubated with shaking at 225 rpm 37 °C for 45 min. The culture was transferred to 200 mL of 2YT supplemented with 50 μg/mL carbenicillin and 20 μg/mL kanamycin) and incubated at 37 °C with shaking at 225 rpm for 18 h. The phages were precipitated as described above, and the resulting phage pellets were resuspended in PBS-T with glycerol (10%, *v*/*v*), separated into 1 mL aliquots, flash frozen with liquid nitrogen, and stored at −80 °C. As required, the phage solution was thawed on ice and precipitated a second time.

After 3 or 4 rounds of selections, spot assays were performed on 96 selectants. Briefly, individual phage colonies were amplified in 96 deep well plates as before. After centrifugation at 3 krpm (1462× *g*), the supernatants were assayed by phage-based ELISA to assess binding to either DJ-1 or the blocking agent, BSA. From these screens, potential DJ-1 binders were isolated and evaluated by Sanger sequencing (Figure S2)

Indirect phage specificity ELISA. A 96-well Nunc Maxisorp plate was coated with 10 μg/mL of either BSA, has, hemoglobin, lysozyme, or *E. coli* supernatant in coating buffer (100 μL/well) and incubated overnight at 4 °C. From this point, the plate was treated identically to that described in Indirect Phage Affinity ELISA.

Direct strep-DPep1-HRP and DJ-1 ELISA. The wells of a 96-well Nunc Maxisorp plate were coated with 10 ug/mL DJ-1 in coating buffer (100 μL) and incubated at 4 °C with shaking at 150 rpm overnight. The coating solution was discarded, and blocking buffer (400 μL) was added to each well. The plate was incubated for 1 h at room temperature with shaking at 150 rpm. Next, a prepared strep-HRP:DPep1 peptide solution (1:4 molar ratio, 38 nM peptide) was serially diluted by a factor of 3 in PBT. The dilutions were added to the appropriate wells (100 μL) and incubated at room temperature with shaking at 150 rpm for 1 h. Then, the plate was treated as described above.

Sandwich strep-DPep1/phage affinity ELISA. A 96-well Nunc Maxisorp plate was coated with 1:24 molar ratio of strep-DPep1 in coating buffer (100 μL) and incubated at 4 °C with shaking at 150 rpm overnight. This solution was discarded and blocking buffer was added to each well (400 μL) for 1 h at room temperature with shaking at 150 rpm. DJ-1 was serially diluted with three-fold dilutions in PBT starting at a 10 μM concentration. The dilutions were added to each well (100 μL) and the plate was incubated at room temperature for 1 h with shaking at 150 rpm. The plate was washed three times and 1 nM of phage selectants were added to each well (100 μL in PBT). The plate was incubated at room temperature for 1 h with shaking at 150 rpm, followed by an additional three washes. Next, 1:5000 anti-M13-HRP was added to each well (100 μL in PBT) and the plate was incubated at room temperature for 30 min with shaking at 150 rpm. Then, the plate was treated as described above.

Sandwich strep-DPep1/phage specificity ELISA. A 96-well Nunc Maxisorp plate was coated with 1:24 molar ratio of strep-DPep1 in coating buffer (100 μL) and incubated at 4 °C with shaking at 150 rpm overnight. This solution was discarded and blocking buffer was added to each well (400 μL) for 1 h at room temperature with shaking at 150 rpm. BSA, HSA, hemoglobin, lysozyme, (10 μM, 100 μL/well), or *E. coli* supernatant in PBT were incubated for 1 h at 4 °C. Then, the plate was treated as described in the Sandwich Strep-DPep1/Phage Affinity ELISA section.

Data analysis. All data and statistics (mean, SEM, *p*-values) were analyzed with GraphPad Prism 9.0. Specificity ELISA data were normalized by division of the DJ-1 signal for each assay type. Analysis of variance (ANOVA) with Sidak’s multiple comparisons was performed to determine significance between DPep3Φ indirect and DPep3Φ sandwich ELISAs. ANOVA with Dunnett’s multiple comparisons was performed to determine significance between DFab1Φ indirect and DFab1Φ sandwich ELISAs.

## Figures and Tables

**Table 1:** Amino acid sequences of DPep1 peptide and PvP selectants.

Peptide	Amino Acid Sequence	Theoretical pI
DPep1	KYRYVCHDVGGTLYCIRDWV	8.03
DPep2	TSYQCHDCGSSLCCVVLPEI	4.35
DPep3	TEIWYLVL	4.00
DPep4	ALTYCYSGPETWVCGQDS	3.67
DPep5	CMKEAMCPV	5.99
DPep6	SLCWNTWFFVCME	4.00
DPep7	DGSYCEYGPVQDACWTNY	3.49
DPep8	PGSYCQYGPMQDACCTNY	3.80
DPep9	SRVFVISCEGPLCTLHTFIA	6.46

**Table 2:** DFab1Φ variable regions.

Fab Site	Amino Acid Sequence
CDRL3	FYYYSGYLF
CDRH1	DFTGDS
CDRH2	SISASGGDTD
CDRH3	WAPHYYAF

## Data Availability

All data are presented in the article and supplement.

## Supplementary Materials

The following supporting information can be downloaded at: https://www.mdpi.com/article/10.3390/v15030807/s1, Figure S1: Additional assays for PvP selections and Strep-DPep1 binding, Figure S2: PvP selectant spot assay results, Table S1: Conditions for PvP peptide selections, Table S2: Proteins used for binding specificity assays, Table S3: Titers, blocking agents, and stringency for PvP Fab selections.
