# Supplementary material for: Phage vs. Phage: Direct Selections of Sandwich Binding Pairs
Source: Viruses. 2023 Mar 22;15(3):807. doi: 10.3390/v15030807 (PMC10057555; doi:10.3390/v15030807)
Supplement: Supplementary file 1 [file viruses-15-00807-s001.zip › viruses-2237658-supplementary.pdf]

SI Appendix:

Phage vs Phage: Direct Selections of Sandwich Binding Pairs for Improved Biomarker Detection

Emily C. Sanders,[a] Alicia M. Santos,[a] Eugene K. Nguyen,[a] Aidan A. Gelston,[a] Sudipta Majumdar,[a] and Gregory A. Weiss*[a,b,c]

Departments of [a]Chemistry, [b]Molecular Biology and Biochemistry, and [c]Pharmaceutical Sciences
University of California, Irvine
Irvine, CA 92697 (USA)
E-mail: gweiss@uci.edu

*To whom correspondence should be addressed.

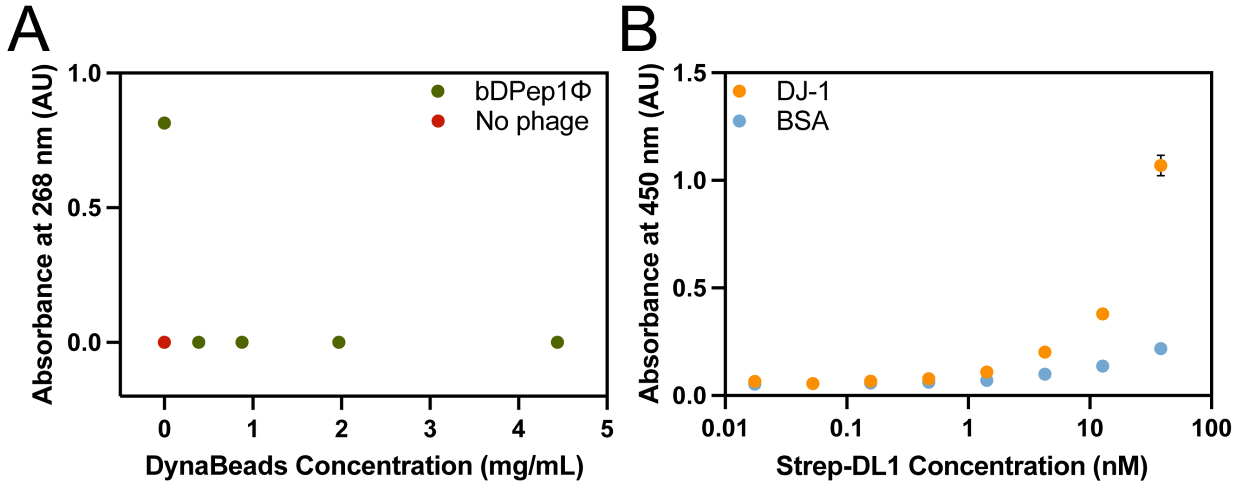

Figure S1. Additional Assays for PvP Selections and Strep-DL1 Binding. A) DynaBeads titration ELISA determined concentration of DynaBeads required for complete pulldown of bDL1Φ. B) The strep-DPep1 complex can bind DJ-1 in a dose-dependent manner.

Table S1. Conditions for PvP peptide selections.

	Titers	Blocking	Washes
**Round 1**	7.9×10^8	Nonfat Milk	3
**Round 2**	3.4×10^5	Bovine serum albumin	6
**Round 3**	2.4×10^5	Human serum albumin	6
**Round 4**	$\sim 1.0 \times 10^6$	Pierce blocking	6

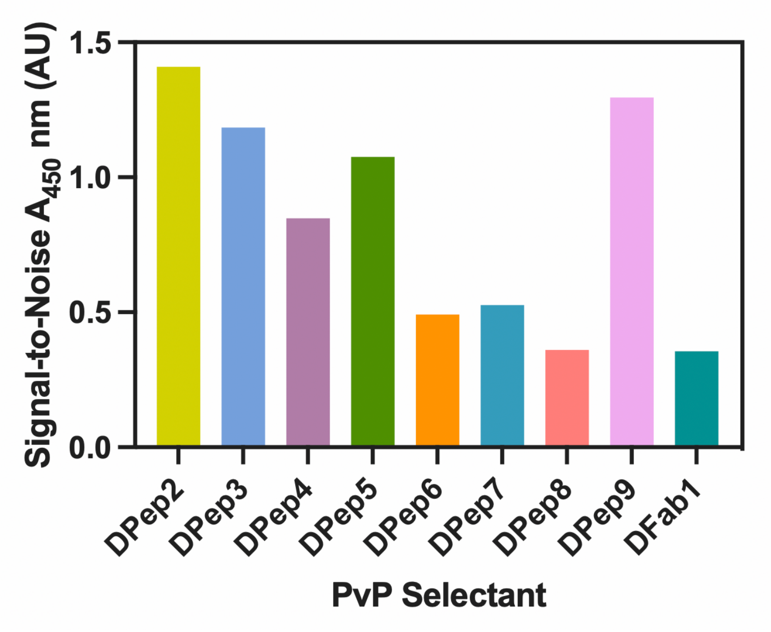

Figure S2. PvP selectant spot assay results. Data are normalized to the signal observed for binding to BSA.

Table S2. Proteins used for binding specificity assays.

Protein	Native Species	Molecular Weight (kDa)	pI
DJ-1	*Homo sapiens*	20.7	6.7
HSA	*Homo sapiens*	66.5	4.7
Hb	*Homo sapiens*	64	6.8
HEWL	*Gallus domesticus*	14.4	11.3
E. coli	*Escherichia coli*	Varies	Varies

Table S3. Titers, blocking agents, and stringency for PvP Fab selections.

	Titers	Blocking	Washes
Round 1	6.7×10^{8}	Nonfat Milk	3
Round 2	3.3×10^{5}	Bovine serum albumin	3
Round 3	6.7×10^{5}	Pierce blocking	6
